# Buccal Mucosa Graft Harvest in Children and Young Adults: Case Series and Harvest Technique

**DOI:** 10.7759/cureus.13884

**Published:** 2021-03-14

**Authors:** Clarice M Brown, Linnea C Fechtner, Philomena M Behar

**Affiliations:** 1 Pediatric Otolaryngology, Emory University School of Medicine, Atlanta, USA; 2 Otolaryngology, University at Buffalo, Buffalo, USA; 3 Otolaryngology, State University of New York at Buffalo, Buffalo, USA; 4 Pediatric Otolaryngology, John R. Oishei Children's Hospital, Buffalo, USA

**Keywords:** urethral stricture, urethroplasty, buccal graft, pediatric reconstructive urology, pediatric otolaryngology

## Abstract

Buccal mucosa is a great choice for urethroplasty for urethral stricture repair because of ease of harvesting, pliability of the graft, and minimal donor site morbidity. These procedures are performed at our institution as a combined case with Pediatric Otolaryngology and Urology. Harvesting buccal mucosal grafts in younger patients is more technically challenging due to limited oral cavity access and smaller area available for tissue harvest, but is able to be performed safely and with limited morbidity with the addition of parotid duct cannulation and use of retraction sutures to the graft harvest technique. This retrospective case series reports harvest technique, outcomes, and complications of children and young adult males undergoing buccal or lower lip mucosal graft harvesting to repair congenital urethral strictures. Outcome measures were perioperative bleeding, trismus, pain, numbness, parotid duct injury and lip deformity. Six patients underwent nine harvest procedures. Technique modifications included application of anterior graft margin stay sutures to help stabilize the graft mucosa and cannulation of the parotid duct with lacrimal probes to avoid duct injury and to maximize graft size. Overall, buccal mucosal graft harvesting is a well-tolerated procedure with minimal complications using proper harvest technique.

## Introduction

Hypospadias is one of the most common congenital defects of the external male genitalia, affecting one in 250 male newborns. This requires surgical repair by a pediatric urologist. The rate of post-repair strictures varies based on surgical technique, ranging from 6-50% [1]. Depending on the size of the stricture, one option for repair of postoperative urethral stricture is a buccal mucosa graft. Buccal and lower lip mucosa are useful for revision urethroplasty because of ease of harvesting, pliability of the graft, and minimal donor site morbidity, the latter of which can be achieved with appropriate harvest technique. Buccal mucosa grafts were first described by Humby in 1941, and intraoral complications can include scarring, trismus, injury to the parotid duct, numbness, and postoperative pain [2]. Harvesting these grafts in younger patients is more technically challenging due to limited oral cavity access and smaller area available for tissue harvest. At our institution, the pediatric otolaryngologist is consulted to assist with harvest of the graft in order to maximize graft size and decrease intraoral complications. We review our experience with oral mucosal graft harvesting in children and young adults and describe our modified harvest technique to assist in maximizing graft size while limiting morbidity. Charts were reviewed of six patients undergoing buccal and/or lower lip mucosal graft harvesting between 2009-2017 at a tertiary care children’s hospital for repair of congenital urethral strictures. The size of the graft was determined during the urethroplasty by the pediatric urologist and the buccal mucosa graft was subsequently harvested by the pediatric otolaryngologist. Outcome measures were perioperative bleeding, trismus, pain, numbness, parotid duct injury, and lip deformity.

## Technical report

**Figure 1:**
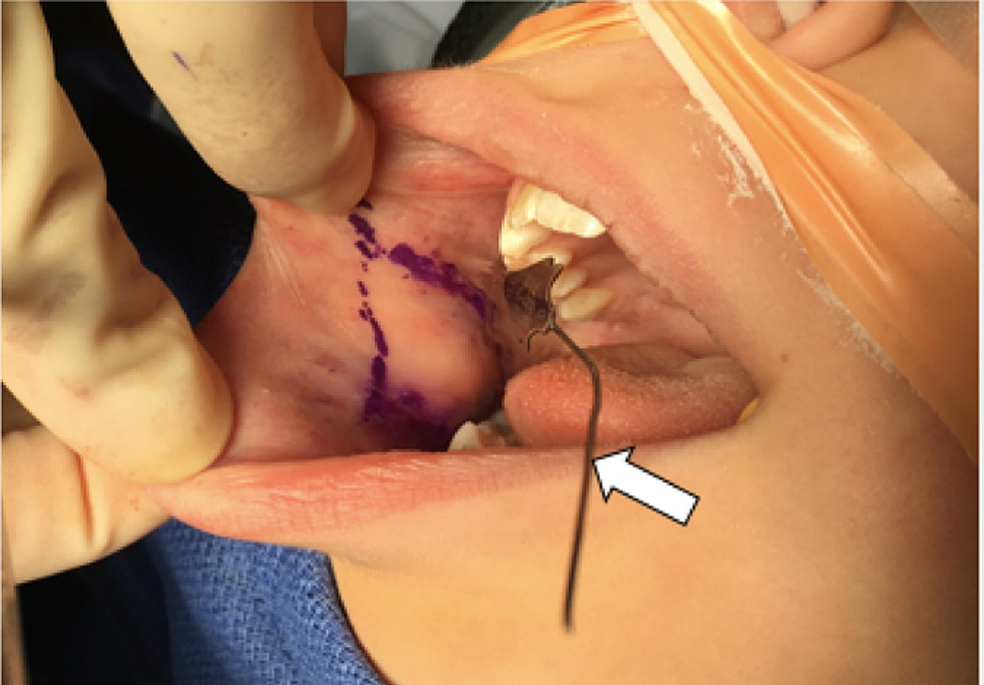
00 lacrimal probe in right Stensen’s duct (white arrow). Right buccal graft outline (purple ink).

After nasotracheal intubation, a bite block was placed and the anterior tongue was retracted with a 2-0 silk suture. The parotid duct was cannulated with a 00 lacrimal probe that was left in place during dissection. Lidocaine 1% with 1:100,000 epinephrine was injected into the submucosa. The graft size was outlined with a marking pen, taking care to mark at least one centimeter (cm) posterior to the oral commissure (Figure 1).

**Figure 2:**
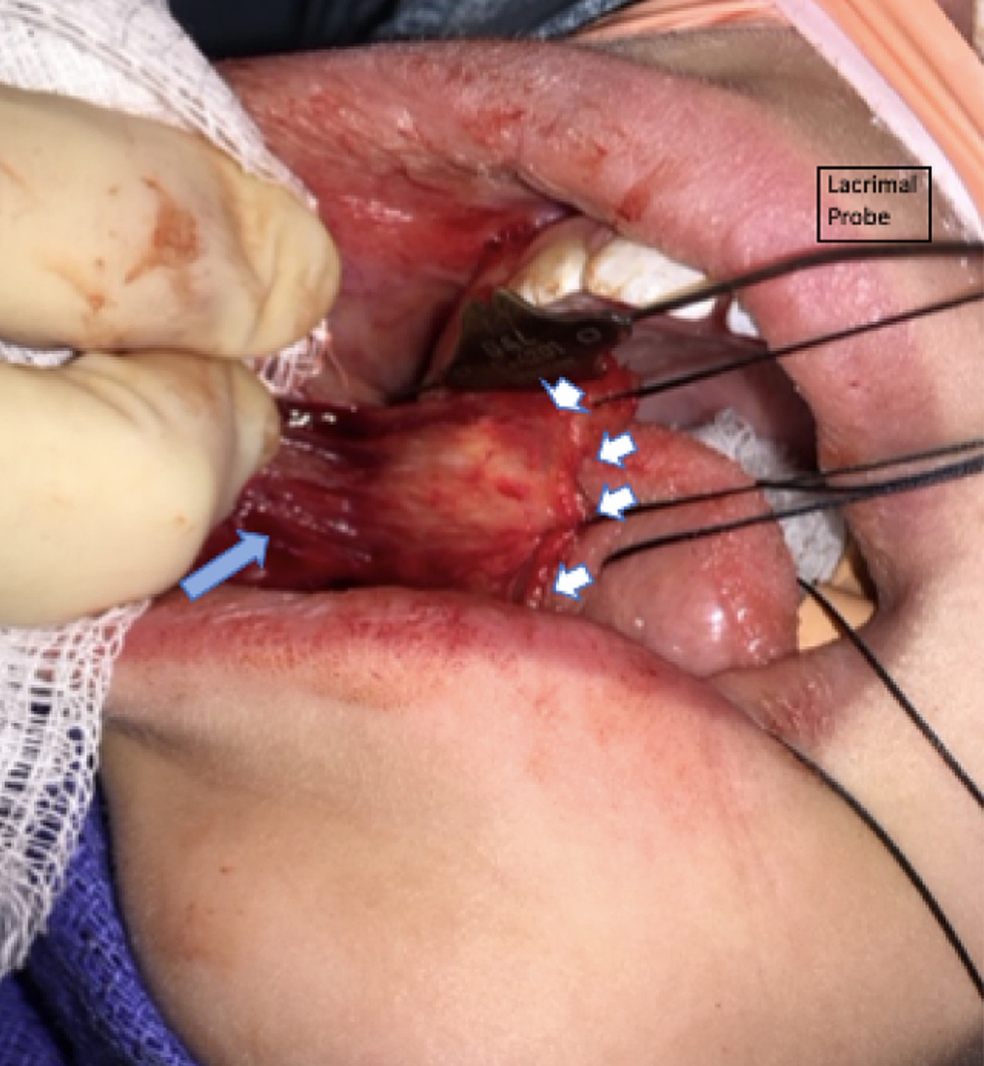
Stabilization of buccal mucosa graft with silk sutures (white arrows). Underlying musculature (blue arrow).

A #15 blade was used to incise through the mucosa and submucosa, and sharp scissors were used to dissect the submucosa from the attached buccinator muscle on the anterior margin. Once freed, 3-0 silk stay sutures were placed on the anterior graft margin to assist with retraction. Dissection continued posteriorly towards the retromolar trigone (Figure 2) and superiorly to 1 cm inferior to the course of the parotid duct, which can be palpated while the probe is in place.

**Figure 3:**
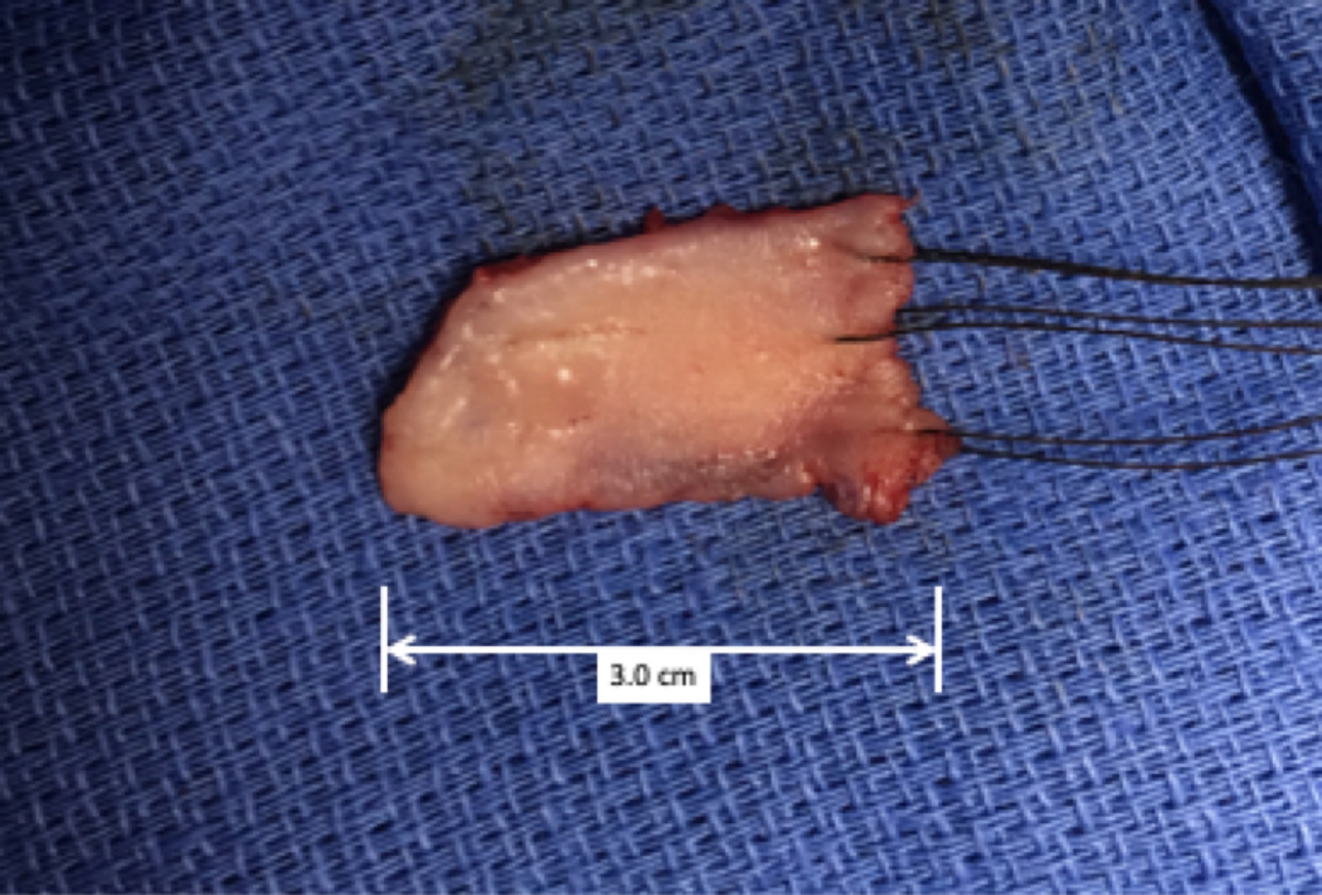
Example of harvested buccal mucosa graft, 3 cm x 2.5 cm, with silk traction sutures attached.

With this technique, a large graft can be obtained, with intraoral margins that spare critical structures, such as the lip margin and the parotid duct. Ideally, the graft should have a width of at least 15-25 mm, and be 2 cm longer than the measured stricture length, as there is approximate 10% contraction over time [3]. Pictured is a 3.0 cm x 2.5 cm graft (Figure 3); graft sizes for the six patients in this case series are included in Table 1.

**Table 1:** Patient demographics and graft characteristics

Patient	Age at Surgery (yr)	Graft Indication	Multiple Grafts	Dimensions of 1^st^graft (cm)	Dimensions of 2^nd^graft (cm)	Laterality of graft
1	17	Urethral stricture	no	3 x 6	n/a	R
2	13	Urethral stricture	no	2 x 2	n/a	R
3	27	Urethral stricture	yes	6 x 5	3 x 2.5	1^st^ R; 2^nd ^L
4	21	Urethral stricture	yes	4.5 x 2.5	3 x 2	1^st ^L; 2^nd^ R
5	9	Urethral stricture	yes	3 x 2.5	4 x 2.5	1^st^ L; 2^nd^ R
6	6	Congenital hypospadias	no	3 x 2.5	n/a	1^st^ R; 2^nd^ L

**Figure 4:**
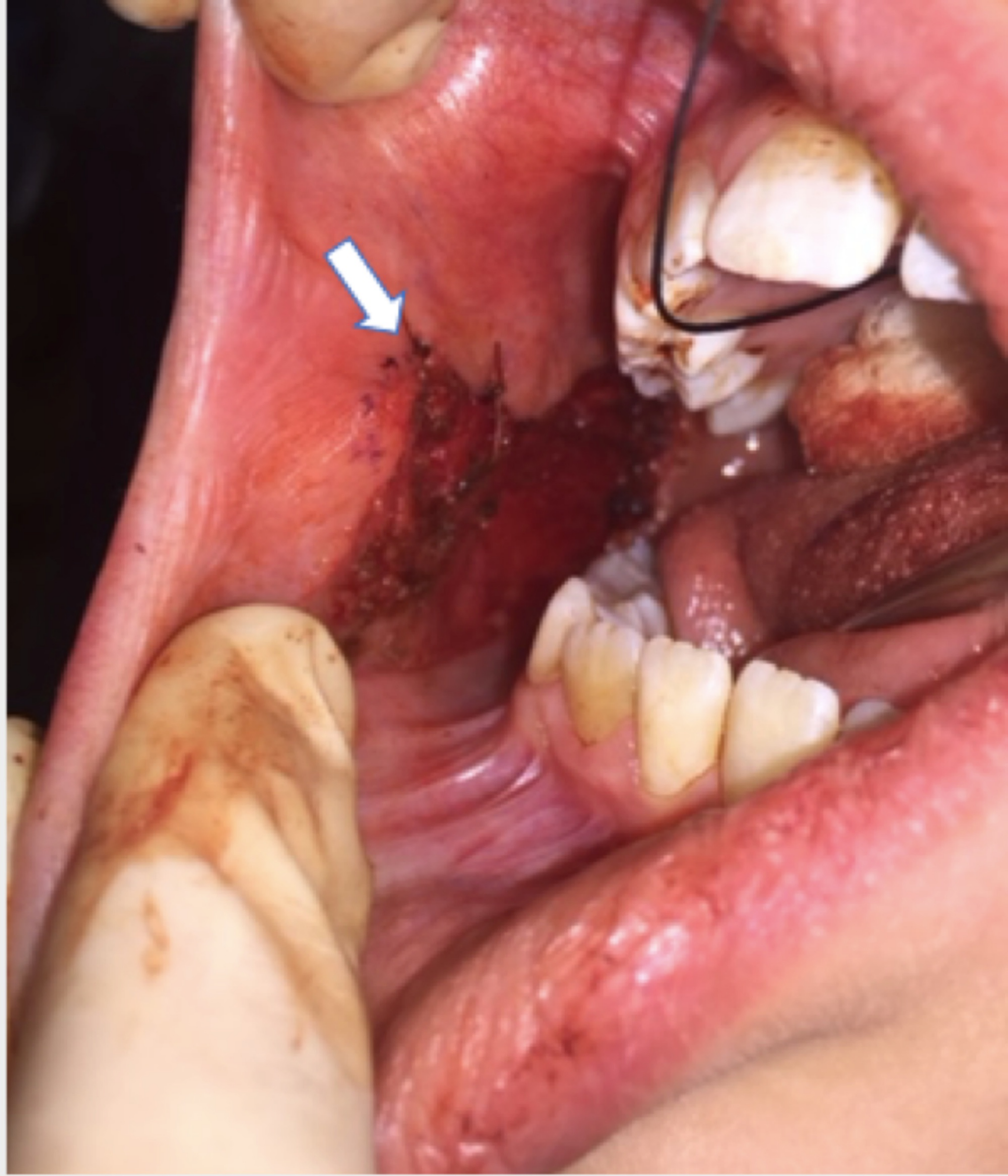
Closure of peripheral wound edges after removal of graft (arrow).

Bipolar cautery at 10 W was used for hemostasis. Simple interrupted sutures using 4-0 chromic gut were applied to the incision margins of the cheek mucosal defect to reduce the size of the wound, the remainder of which closes by secondary intention (Figure 4).

**Figure 5:**
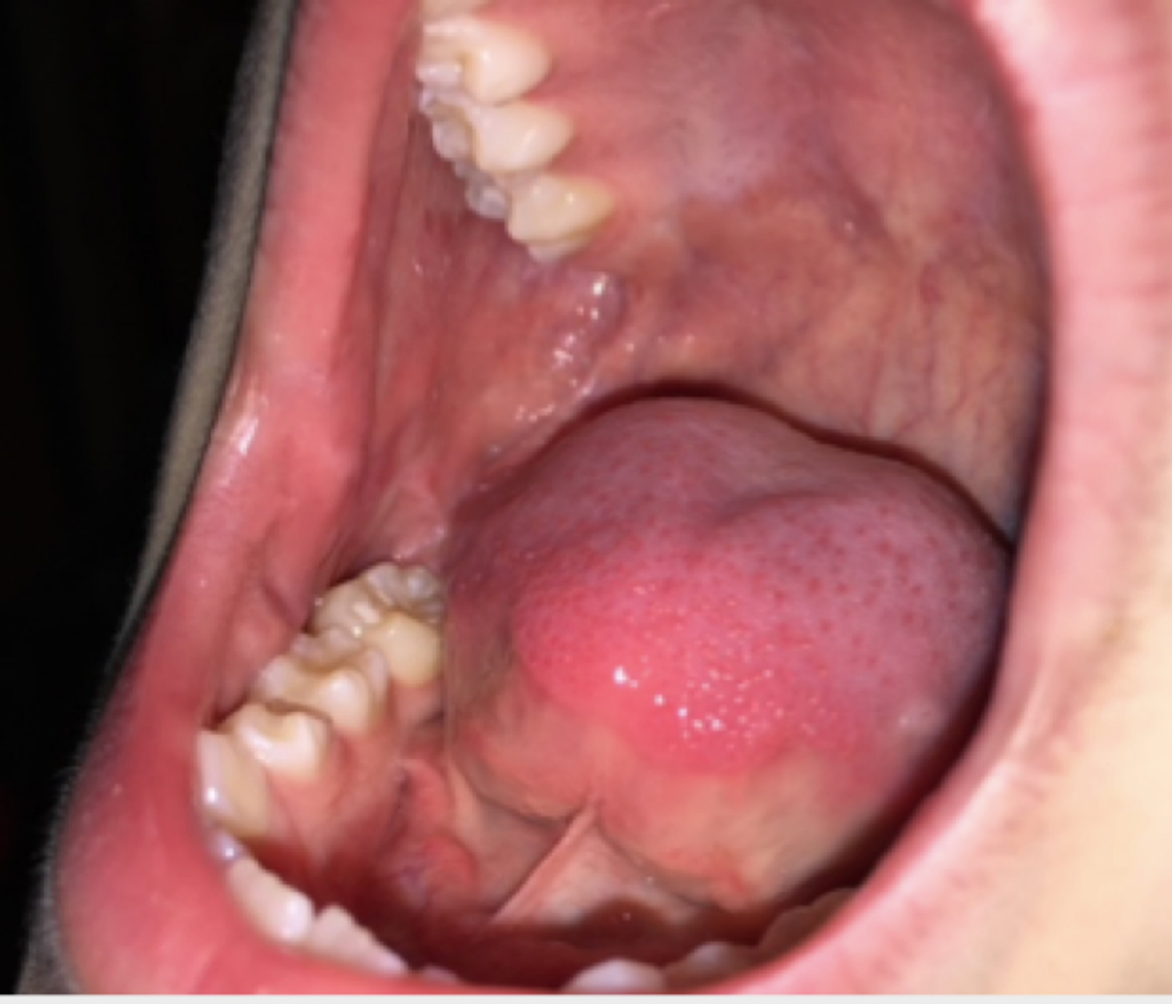
Healed right buccal mucosa at six weeks.

Clear liquid diet was started on the day of surgery and advanced as tolerated. Saline oral rinses were performed after meals for one week. Patients were evaluated six weeks post operatively, and the buccal donor site appeared well healed (Figure 5).

## Discussion

A total of six male patients underwent buccal mucosa graft harvesting for urethroplasty over an eight-year period. Two patients had multiple separate buccal mucosa graft harvesting procedures over the eight-year period for recurrent stricture. The mean patient age was 13 years (range 6-29 years). Graft length and width ranged from: 2 to 8 cm and 2 to 3.5 cm, respectively. The average time to follow-up was 15 days after surgery (Table 1).

At the post-operative visit, 2/6 (33%) of the patients reported mild tenderness. The remainder of the patients reported no pain. No patient experienced postoperative bleeding; 1/6 (17%) patients reported temporary numbness; 1/6 (17%) patient reported temporary trismus. The patient who reported trismus was a 27-year-old smoker and also had a benign leukoplastic lesion on the contralateral buccal mucosa that was biopsied at the time of graft harvesting. His trismus resolved three months post operatively with normal interincisal opening on physical exam. No patients reported or had evidence of parotid duct injury or parotitis. One patient had a combined labial and buccal graft in order to obtain a longer graft.

Our results show there is minimal morbidity with our modified technique for buccal mucosa graft harvesting in pediatric and young adult patients. The two main technique modifications made to safely maximize graft size and avoid graft and harvest bed injury were parotid duct cannulation and stay suture placement, which have not been described in prior studies.

The parotid duct was cannulated before injection and marking of the graft. To our knowledge, no prior study has described cannulation of the parotid duct prior to harvesting. One study described parotid duct injury with subsequent ductal stenosis in two patients after buccal mucosal graft harvesting [4]. Two of 256 patients presented with painful swelling over the parotid region. Both patients were treated by exploration and stenting of the duct. No other studies reported parotid duct injury. If graft harvesting is done by surgeons unfamiliar with oral cavity anatomy, cannulation of the duct helps prevent accidental injury while maximizing graft harvest size.

Second, the application of stay sutures to the anterior margin of the graft during harvest is also important. This helps in manipulation of the graft during dissection and avoids damage to the graft with forceps during harvesting. It is also helpful in taking a thin, pliable graft allowing for excellent traction to separate the submucosa from the buccal musculature. Avoidance of deep dissection may be important for better healing, less pain and scarring as well as avoidance of sensory nerve damage.

Fabbroni et al. described a similar technique where the graft was marked out to avoid the parotid duct however, the parotid duct was not cannulated [5]. In addition, 1 cm cuff of mucosa was maintained posterior to labial commissure. After the graft was removed, the mucosal edges were sutured to the underlying muscles to reduce the size of the harvest bed. One patient in their study also experienced postoperative trismus due to submucosal scar bands. The trismus did not self-resolve and the patient required scar band release. Another study was complicated by scarring and wound contracture. Buccal graft was harvested by the oral maxillofacial team and the patient eventually required surgical revision via Z-plasty [6].

Complete closure of donor sites is controversial and may contribute to scar formation and increased perioperative pain. All of our patients had complete mucosalization of the donor site at their six-week post-operative appointment. The donor site healed well with secondary intention. Placing absorbable, interrupted sutures along the graft harvest wound edges to reduce the size of the defect seems to help reduce the defect size. All patients were able to tolerate regular diet within 24 hours after surgery, despite two patients reporting mild tenderness. Wong et al. published a prospective study comparing primary closure versus secondary intention in patients who underwent buccal mucosal graft harvesting [7]. Patients who were allowed to heal by secondary intention had significantly higher pain scores than patients who underwent primary closure. Patients within the secondary intention group also had more discomfort with an oral diet. However, there was no difference in numbness or tightness between the two groups.

Buccal mucosa is a very useful graft for reconstruction for urethral stricture, and is harvested at our institution by the pediatric otolaryngologist for use by the pediatric urologist. This allows for a two-team approach to this reconstruction, which likely allows for decreased operative time. However, we were unable to fully examine this in our study as we did not have any cases where the graft was not harvested by otolaryngology.

## Conclusions

Buccal mucosa is a very useful graft for reconstruction for urethral stricture, and is harvested at our institution by the pediatric otolaryngologist for use by the pediatric urologist. Although parotid duct injury is a rare complication, it can cause significant morbidity. This can be avoided with no additional morbidity by duct cannulation and palpation during graft marking and elevation. The addition of anterior graft margin stay sutures is helpful in harvesting a thin, even graft and helps avoid trauma to the graft and to the buccal musculature, which helps reduce long-term complications of scarring and trismus. Overall, buccal mucosal graft harvesting is a well-tolerated procedure with minimal complications using proper harvest technique.
